# Trait prioritization and genotype selection for heat stress tolerance in wheat *via* structural equation modeling and principal component analysis (PCA)

**DOI:** 10.7717/peerj.21335

**Published:** 2026-06-23

**Authors:** Irum Khan, Muhammad Kashif Naeem, Mehraj Abbasov, Muhammad Ramzan Khan, Zi Jin Zhang, Jing Chen, Muhammad Sajjad

**Affiliations:** 1 Department of Biosciences, COMSATS University Islamabad, Islamabad, Islamabad Capital Territory, Pakistan; 2 Chengdu Institute of Biology, Chinese Academy of Sciences, Chengdu, China; 3 National Institute for Genomics and Advanced Biotechnology (NIGAB), National Agricultural Research Center (NARC), Islamabad, Pakistan; 4 School of Agricultural and Food Sciences, ADA University, Baku, Baku City, Azerbaijan

## Abstract

Terminal heat stress is a major limitation to wheat productivity, especially during the reproductive stage. This paper examines the genetic variation and correlations of heat tolerance traits in wheat using structural equation modeling (SEM) as a novel approach to reveal causal relationships among key reproductive traits. Genetic diversity was evaluated in 200 spring wheat genotypes grown under optimal and heat-stressed environments across two consecutive growing seasons. The traits studied included pollen viability (PV), grain number per spike (GpS), spikelet fertility (SpF), fertile florets per spikelet (FFpSp), grain filling duration (GFD), and anther length (AL). Notably, GpS and AL showed high heritability and genetic advancement in both environments, indicating a strong genetic influence and good potential for selection. SEM analysis revealed that SpF was the major direct contributor to GpS under normal conditions, whereas PV was the primary contributor under heat stress—an association not previously well documented in the literature. These findings were supported by principal component analysis (PCA), which showed that the first and second principal components explained 53.1% and 56.3% of the total variance in the first and second years, respectively. Trait correlations differed between environments: under normal conditions, GFD and GpS were closely aligned, while under heat stress, PV, SpF, and FFpSp clustered together, indicating a coordinated physiological response to stress. The results emphasize PV as a critical trait for selecting heat-tolerant wheat lines. Furthermore, three genotypes—Chenab-70, Pak-81, and Frontana—showed consistent tolerance across environments, providing valuable genetic resources for breeding climate-resilient wheat. This study presents a comprehensive analytic model integrating SEM and PCA to improve the accuracy of selecting physiological traits in wheat breeding under terminal heat stress.

## Introduction

Wheat yields are significantly affected by heat stress, largely due to the sensitivity of reproductive development to elevated temperatures (Bala, Asthir & Bains, 2014; Sharma et al., 2019; Ullah et al., 2020; Kumar et al., 2023; Vedi & Pandey, 2024; (Khanzada et al., 2025). At the global scale, ongoing climate change has intensified temperature extremes, leading to substantial declines in wheat productivity (Araus et al., 2002; Lesk, Rowhani & Ramankutty, 2016; Shenoda et al., 2021; Nyaupane et al., 2024). By the end of the 21st century, temperatures are projected to increase by 1–4 °C, potentially reducing wheat yield by 4–6% (Driedonks, Rieu & Vriezen, 2016; Liu et al., 2016). The optimum temperature for spring wheat during the anthesis and grain-filling stages ranges between 12 °C and 22 °C (Farooq et al., 2011a; Farooq et al., 2011b; Farooq et al., 2009); however, temperatures above this range cause significant reductions in wheat yield (Kaur & Behl, 2010; Khan et al., 2015). An increase of 1–2 °C above the optimum temprature adversely affects key agronomic traits, including grain-filling duration, pollen viability, and spike fertility, contributing to yield losses (Talukder, McDonald & Gill, 2014; Nasehzadeh & Ellis, 2017; Zhao et al., 2022; Tomás et al., 2020; Masthigowda et al., 2022). The impact is particularly critical in South Asia, where wheat is a major food crop and rising temperatures have already reduced yield stability (Hussain et al., 2023; Shahzad, Akram & Ashraf, 2019; Uzair et al., 2025).

In Pakistan, field-based studies have confirmed similar reductions in grain-filling duration and spike fertility under heat stress (Shahzad, Akram & Ashraf, 2019; Uzair et al., 2025). A moderate temperature increase of ∼1 °C may reduce wheat yield by about 7%, although this response varies among genotypes (Khan et al., 2020). Furthermore, elevated night temperatures of 4–5 °C can shorten the grain-filling period by 3–7 days, leading to premature maturity and accelerated leaf senescence (Prasad et al., 2008; Dias & Lidon, 2009; Narayanan, 2018; Schittenhelm et al., 2020).

Several studies have confirmed the negative impacts of heat stress on a range of wheat morpho-physiological and yield-related traits (Sattar et al., 2010; Hamam, Khaled & Zakaria, 2015; Wang et al., 2018; Bheemanahalli et al., 2019; Nizamani et al., 2020; Riaz et al., 2021). Despite this, relatively few studies from Pakistan and neighboring countries have focused on multi-trait analyses of heat tolerance, especially using advanced multivariate approaches such as structural equation modeling (SEM) and principal component analysis (PCA) (Khan et al., 2020; Telfer et al., 2021).

Identifying key adaptive traits under heat stress is vital for breeding new wheat genotypes with improved tolerance across heat stress environments (Fu et al., 2023; Mohammadi & Amri, 2022). Additionally, evaluating the relationships between morphophysiological traits under heat stress is crucial for effective selection and the improvement of breeding programs (Chitralekha et al., 2018). The substantial genetic diversity present within wheat offers breeders valuable opportunities to select and develop cultivars with enhanced tolerance to high-temperature conditions (Sunita et al., 2017; Dhami et al., 2018; Mahdavi et al., 2022). Nevertheless, complex environmental interactions with these agronomic traits continue to challenge breeding efforts aimed at achieving stable, high-yielding wheat under thermal stress (Kamrani, Hoseini & Ebadollahi, 2018; Poudel et al., 2020; Sharma et al., 2019).

Breeders have commonly assessed the effects of heat stress through the evaluation of multiple yield-related traits, such as plant height, phenological stages (days to heading and maturity), spike and spikelet characteristics, grains per spike, thousand-kernel weight, grain yield per spike, biological yield, and harvest index (Saini & Aspinall, 1982; Din et al., 2010; Cai et al., 2016; Sharma et al., 2016; Rezaei et al., 2018; Xu et al., 2022). Many high-yielding cultivars are losing their potential due to adverse climatic changes (Pequeno et al., 2021; Elahi et al., 2022; Riedesel et al., 2024; Sahin, 2025). Despite extensive research on heat stress and other factors independently, little is known about their interaction and combined impact on grain yield (Telfer et al., 2018). Moreover, few studies have investigated the effects of extreme heat stress (>35 °C) on wheat yield-related characteristics and pollen viability during the spike initiation stage in the field (Masthigowda et al., 2022). Research on these topics has been limited to only a few genotypes (Oliveira et al., 2020; Poude et al., 2020).

Importantly, there remains a gap in large-scale field evaluations of diverse wheat germplasm under heat stress in South Asia, where breeding for climate resilience is an urgent priority. Previous studies have largely focused on small genotype sets or single traits, while few have integrated multi-trait SEM and PCA analyses to examine the direct and indirect associations of heat stress on yield-related traits. Herein, we conducted a comprehensive two-year field trial using 200 genotypes of spring wheat under both normal and heat-stressed conditions. This study focused on identifying dependable screening traits for evaluating terminal heat tolerance in wheat, along with recognizing genotypes that consistently perform well under heat stress for those traits. While pollen viability is a well-established screening trait for heat tolerance, this study provides further support for its established importance by integrating SEM and PCA approaches across a large panel of 200 genotypes grown under South Asian field conditions, enabling comparative analysis of trait pathways across years and environments. By integrating SEM and PCA approaches, our work provides a comprehensive multivariate analytical framework that extends previous studies, offering a robust methodological framework to understand complex trait interactions under South Asian field conditions. The findings provide valuable insight into the traits and genotypes associated with heat tolerance, offering a practical foundation for breeding wheat varieties with improved resilience to elevated temperatures.

## Materials and Methods

### Field experiment and experimental design

The field experiments were conducted at the experimental site of National Institute for Genomics and Advanced Biotechnology, National Agricultural Research Center (NARC), Islamabad, Pakistan (33.67°N, 73.13°E), over two consecutive wheat-growing seasons (2020–2021 and 2021–2022). A total of 200 spring wheat genotypes were evaluated using a randomized complete block design (RCBD) with three replications to minimize spatial field variability and ensure reliable detection of genotypic differences. Seeds were sown using a wheat planter in plots measuring 1.2 m × 3 m, each consisting of six rows spaced 20 cm apart. Prior to sowing, the field was cultivated to a depth of approximately 15 cm and leveled to ensure uniform soil conditions. A seeding density of 200 seeds m^−2^ was maintained. Fertilizer was applied following recommended agronomic practices, with NPK (20:20:20) at 150 kg ha^−1^ applied at sowing and urea (46% N) at 100 kg ha^−1^ applied at the tillering stage. Irrigation was supplied at 7-day intervals to maintain soil moisture near field capacity. Weeds were removed manually every two weeks, and no chemical pesticides were used throughout the growing period.

To further justify the experimental design, each replication constituted a complete block containing all 200 genotypes to minimize environmental heterogeneity across the experimental area. Plots within each block were arranged systematically in rows with uniform spacing and were randomized prior to sowing to avoid positional bias. Field preparation, including leveling, uniform fertilizer application, and consistent irrigation scheduling, was undertaken to reduce potential gradients in soil fertility and moisture. Border rows were maintained around experimental plots to limit edge effects. Although incomplete block designs such as alpha-lattice can be advantageous for large genotype sets, RCBD was selected due to the relatively uniform field conditions, manageable plot size, and logistical simplicity for conducting multi-year heat stress field trials. These measures collectively helped ensure that observed phenotypic differences primarily reflected genotypic variation rather than field spatial effects.

The experimental soil was classified as moderately calcareous clay loam to silty clay loam with good drainage, an alkaline pH (∼7.5), and moderate organic matter content (∼0.8%). Climatic data, including monthly mean temperature and rainfall during the wheat growing seasons (2020–2022), were obtained from the Pakistan Meteorological Department (PMD, 2022). During the cropping period, average monthly maximum temperatures ranged from approximately 20 to 35 °C, while minimum temperatures ranged from about 1 to 10 °C. The site received an average annual rainfall of approximately 1200 mm.

### Heat-stress treatment and microclimate monitoring

Heat stress was imposed by covering experimental plots with transparent plastic sheets during the critical reproductive period, from spike initiation (Zadoks 50) to physiological maturity (Zadoks 91). Control (normal) plots were left uncovered. To validate that the intended heat stress was achieved and to control for potential confounding microclimate effects, we conducted continuous microclimate monitoring in both treatments.

Air temperature and relative humidity were monitored using digital temperature and humidity sensors (HTC-2 Digital Room Hygrometer Thermometer Clock LCD Indoor/Outdoor Temperature Humidity Meter with sensor). The sensors were installed at approximately 1.5 m above ground level within the crop canopy and connected to data loggers programmed to record measurements at 10 min intervals throughout the covering period. Three replicate sensors were deployed per treatment to ensure spatial representation across the field. Canopy temperature was measured at midday using a handheld infrared thermometer (Testo 830 T2), and soil temperature and volumetric soil moisture were monitored at 10 cm depth using UbiBot RS485 soil probes. These instrumentation procedures follow established field microclimate monitoring protocols (Lobell & Asner, 2003; Maia et al., 2019).

To improve transparency and clarity of stress imposition, summary statistics of the microclimate data showed that covered plots consistently experienced approximately 2–4 °C higher air temperature and slightly lower relative humidity (mean decrease ∼3%) compared with control plots during the reproductive and grain filling periods, confirming that the stress treatment produced a sustained thermal differential. Canopy and soil temperatures were also elevated under the plastic covers. Importantly, light (Photosynthetically Active Radiation (PAR)) reduction under the plastic sheets was minimal (<5%), and airflow was sufficient to prevent excessive condensation, ensuring that observed physiological and yield responses were primarily attributable to elevated temperature rather than unintended microclimate artifacts. The microclimate data are presented in Table 1 for clear comparison between treatments, showing mean ± standard deviation (SD) values for air temperature, relative humidity, canopy temperature, soil temperature, and soil moisture under both treatments.

**Table 1:** Microclimate conditions under normal and heat-stress treatments during the 2020–2021 and 2021–2022 growing seasons. Values are presented as mean ± standard deviation (SD) for the 2020–2021 and 2021–2022 growing seasons. Air temperature and relative humidity were recorded at 10-min intervals. Canopy temperature was measured at midday, soil temperature at 10 cm depth, and soil moisture as volumetric water content. In both growing seasons, the heat-stress treatment maintained consistently higher temperature conditions compared with the normal.

**Season**	**Parameter**	**Normal (mean ± SD)**	**Heat-stress (mean ± SD)**	**Notes**
2020–2021	Air temperature (°C)	28 ± 2	30–32 ± 2	Recorded at 10-min intervals within canopy
2020–2021	Relative humidity (%)	65 ± 5	62 ± 4	Recorded at 10-min intervals
2020–2021	Canopy temperature (°C)	28 ± 1.5	31 ± 1.8	Midday measurements during anthesis–grain filling
2020–2021	Soil temperature (°C)	22 ± 1	24 ± 1.2	Measured at 10 cm soil depth
2020–2021	Soil moisture (%)	25 ± 3	24 ± 3	Volumetric water content; similar irrigation maintained
2021–2022	Air temperature (°C)	27 ± 2	29–31 ± 2	Recorded at 10-min intervals within canopy
2021–2022	Relative humidity (%)	66 ± 5	63 ± 4	Recorded at 10-min intervals
2021–2022	Canopy temperature (° C)	27.5 ± 1.5	30.5 ± 1.7	Midday measurements during anthesis–grain filling
2021–2022	Soil temperature (°C)	21.5 ± 1	23.5 ± 1.1	Measured at 10 cm soil depth
2021–2022	Soil moisture (%)	26 ± 3	25 ± 3	Volumetric water content; irrigation maintained

### Heat-stress duration and timing

The heat-stress treatment was applied continuously from spike initiation (Zadoks 50) to physiological maturity (Zadoks 91), lasting approximately 80 days in both seasons. In the 2020–2021 season, this period corresponded to (12 February–20 May), and in 2021–2022 to (28 February–18 May). The plastic covering remained in place for the entire duration of this period to ensure continuous exposure to elevated temperatures during the reproductive and grain filling stages. Normal plots were left uncovered during the same calendar period.

The plastic sheets were installed above the crop canopy with regular openings to maintain air circulation and minimize excessive humidity accumulation. Although these measures were implemented, a slight increase in localized humidity may still have occurred and is therefore acknowledged as a potential limitation of the study. The heat-stress treatment produced an approximate increase of 2–4 °C in both air and canopy temperature compared with ambient conditions, indicating that the imposed stress corresponded to moderate rather than extreme terminal heat stress (>30 °C). This treatment was designed to simulate terminal heat conditions typical of Islamabad, where temperatures during the reproductive stage generally rise moderately above ambient levels. Consequently, the observed trait responses can largely be attributed to elevated temperature rather than confounding effects from altered light, humidity, or other microclimatic factors.

### Data collection

Data for most agronomic and reproductive traits were recorded at physiological maturity. For each genotype, three randomly selected plants per plot were sampled, and observations were primarily taken from the main tiller to minimize within-plant variability. Trait values from sampled plants were averaged for statistical analysis. Because the 200 wheat genotypes differed in phenology, anthesis and maturity observations were recorded separately for each genotype to avoid bias associated with differences in developmental timing.

Grain filling duration (GFD) was calculated as the number of days from anthesis to physiological maturity (Pržulj & Mladenov, 1999). Anthesis and maturity dates were recorded individually for each genotype. Grain number per spike (GpS) was determined at physiological maturity by counting grains from one representative main spike per sampled plant (three spikes per plot). Mean grain number per spike was calculated across the sampled plants (Xu et al., 2022). Spikelet fertility (SpF) was assessed at physiological maturity based on seed set. Fertile and total spikelets were counted from the main spike of each sampled plant. Spikelet fertility (%) was calculated as:

Spikelet fertility (%) = (Number of fertilespikelets/Total number ofspikeletsper spike) × 100 (Liu et al., 2006).

Fertile florets per spikelet (FFpSp) were evaluated on the main tiller spikes. The central spikelet of each selected spike was marked at anthesis using cotton thread. At physiological maturity, florets were examined for grain presence, and both partially and fully filled florets were counted to estimate fertile florets per spikelet (Prasad Djanaguiraman, 2014). Anther length (AL) was measured at anthesis using three biological replicates per genotype under both normal and heat stress conditions. Fully developed anthers were collected from the central spikelets of the main tiller spikes, and their lengths were measured using a calibrated scale (Atashi-Rang & Lucken, 1977).

### Pollen viability test

Pollen viability was assessed using a modified Alexander test (Dafni & Firmage, 2000) following anthesis. Fresh anthers were collected from each genotype and stored at 4 °C for no longer than 30 min prior to staining to minimize viability loss. Storage conditions and duration were identical across all samples. For each genotype, pollen from at least three biological replicates was stained, and ∼30–50 pollen grains per sample were counted under an Olympus compound microscope. Darkly stained grains were considered fertile, and lightly stained grains sterile. Pollen viability (%) was calculated as the ratio of fertile to total pollen grains (Prasad et al., 2008). To ensure reliability, counts were repeated for randomly selected samples under standardized microscopic conditions. All procedures were performed within the same timeframe to minimize temporal or environmental variability.

### Statistical analysis

Heat susceptibility index (HSI) for yield-related parameters, including pollen viability (PV), grain number per spike (GpS), grain filling duration (GFD), spikelet fertility (SpF), fertile florets per spikelet (FFpSp), and anther length (AL) was calculated using the following formula suggested by Fischer & Maurer (1976). Based on HSI, genotypes were grouped into three classes: tolerant (HSI < 0.5), moderately tolerant (HSI 0.5–0.99), and susceptible (HSI > 1.0).

\begin{eqnarray*}\mathrm{HSI}=[1-(\mathrm{X}\mathrm{_}\mathrm{stress}/\mathrm{X}\mathrm{_}\mathrm{normal})]/[1-(\overline{\mathrm{X}}\mathrm{_}\mathrm{stress}/\overline{\mathrm{X}}\mathrm{_}\mathrm{normal})]. \end{eqnarray*}

Analysis of variance (ANOVA) was applied to evaluate the effects of genotype (G), treatment (control *vs* heat stress), year, and their interaction (genotype × treatment) on all measured traits (Gomez & Gomez, 1984). A factorial ANOVA model was implemented using R software (version 4.5.2). In this model, genotype and treatment were treated as fixed factors, while year was treated as a random factor. The genotype × treatment (G × T) interaction was included in the model, as presented in Table 2.

**Table 2:** Analysis of variance (ANOVA) for the studied traits of 200 wheat genotypes evaluated under normal and heat stress conditions during 2020–2022. Analysis of variance (ANOVA) for reproductive and yield-related traits of wheat evaluated under normal and heat stress conditions across two growing seasons (2020–2021 and 2021–2022). The table reports mean square values for pollen viability (PV), grain number per spike (GpS), spikelet fertility (SpF), fertile florets per spikelet (FFpSp), grain filling duration (GFD), and anther length (AL), analyzed across environments and seasons. Sources of variation include genotype, environment (normal vs. heat stress), season, and their interactions (where applicable).

Mean sum of squares							
Source of variation	DF	PV	GpS	GFD	SpF	FFpSp	AL
Treatment	1	40,809^**^	161,360^**^	79,212^**^	113,691^**^	4,797^**^	330^**^
Genotypes	199	13,860^**^	417^*^	85^*^	236^**^	3^*^	1.7^*^
Year	1	172^**^	14,706^**^	10,037^**^	14,727^**^	487^**^	47.08^**^
Genotypes:Treatment	199	100^**^	105^**^	23^**^	168^**^	2^*^	0.4^*^
Residuals	1,999	36	21	11	41	2	0.31

**Notes.**

Statistical significance was determined using ANOVA, with **, and * indicating significance at *p* < 0.001, *p* < 0.01, and *p* < 0.05, respectively; ns denotes non-significant effects.

Phenotypic coefficient of variation (PCV), genotypic coefficient of variation (GCV), broad-sense heritability (${h}_{\mathrm{bs}}^{2}$) and genetic advance (GA) were calculated using percent pollen viability values under normal and stress conditions. The following formulas (Allard, 1960) were used in Microsoft Excel 2016 to calculate these parameters:

\begin{eqnarray*}\mathrm{PCV}~(\%)=\surd ({\sigma }^{2}\mathrm{p})/\mathrm{X}\overline{\times }100\nonumber\\\displaystyle \mathrm{GCV}~(\%)=\surd ({\sigma }^{2}\mathrm{g})/\mathrm{X}\overline{\times }100\nonumber\\\displaystyle {\mathrm{h}}^{2}\mathrm{bs}~(\%)=({\sigma }^{2}\mathrm{g}/{\sigma }^{2}\mathrm{p})\times 100\nonumber\\\displaystyle \mathrm{GA}=\mathrm{k}\times \surd ({\sigma }^{2}\mathrm{p})\times {\mathrm{h}}^{2}\mathrm{bs} \end{eqnarray*}

where:

*σ*^2^p = phenotypic variance

*σ*^2^g = genotypic variance

$\bar {X}$ = mean value

k (constant) = 2.06 at 5% selection intensity.

Correlation analysis was performed using the corrplot package using R software (R Core Team, 2024). These predictors were further incorporated into a structural equation model (SEM) to estimate direct and indirect associations, with model specification informed by theoretical expectations and prior studies (Grace, 2006). Structural equation modeling (SEM) was conducted using the lavaan package on pooled data from both experimental years and treatment conditions to quantify direct and indirect associations among pollen viability, anther length, fertile florets per spikelet, spikelet fertility, grain number per spike, and grain filling duration, with model structure guided by theoretical expectations and prior studies (Grace, 2006). SEM followed covariance-based modeling procedures (Grace, 2006; Kline, 2016). Model fit was evaluated using multiple goodness-of-fit indices, including the chi-square to degrees-of-freedom ratio (*χ*^2^/df), comparative fit index (CFI), Tucker–Lewis index (TLI), root mean square error of approximation (RMSEA), and standardized root mean square residual (SRMR). Threshold values for acceptable model fit were *χ*^2^/df < 3.0, CFI and TLI > 0.90, RMSEA < 0.06, and SRMR < 0.08 (Hu & Bentler, 1999; Kline, 2016). The proportion of variance explained (R^2^) was calculated for endogenous variables to assess the explanatory strength of the modeled pathway.

Principal component analysis (PCA) was conducted on standardized trait data to generate biplots and component summaries, visualizing multivariate trait variation and genotype responses under contrasting temperature conditions.

### Results and Discussions

### Microclimate validation under heat stress

Microclimate monitoring confirmed that heat-stress plots experienced 2–4 °C higher air temperatures and slightly lower relative humidity (∼3%) compared with control plots during the reproductive and grain-filling periods (Table 1). Canopy and soil temperatures were also elevated, while soil moisture and light (PAR) were minimally affected (<5% reduction), and airflow under the plastic covers was sufficient to prevent condensation. These results validate that the plastic coverings effectively imposed sustained heat stress. The observed thermal differential aligns with previous studies showing that transparent coverings can increase canopy temperature without substantially affecting humidity or light (Lobell & Asner, 2003; Maia et al., 2019) and indicates that the physiological and yield responses measured in this study can be primarily attributed to elevated temperature rather than unintended microclimate artifacts (Sharma, Chapagain & Marasini, 2020;Farooq et al., 2011a; Farooq et al., 2011b; Farooq et al., 2011c).

### Genetic variability and genotype × heat stress responses

Analysis of variance revealed highly significant differences among wheat genotypes for all evaluated traits under both normal and heat stress conditions, demonstrating substantial genetic variability within the panel (Table 2). The highly significant genotype × treatment interaction across traits indicates differential genotypic responses to thermal regimes, underscoring the complexity of heat stress tolerance mechanisms in wheat. Such interaction effects highlight the importance of multi-environment evaluation when selecting heat-resilient genotypes (Majeed et al., 2023; Mahdavi et al., 2022).

### Impact of heat stress on reproductive and yield-related traits

Heat stress significantly reduced pollen viability (PV), grains per spike (GpS), grain filling duration (GFD), spikelet fertility (SpF), fertile florets per spikelet (FFpSp), and anther length (AL) across all 200 genotypes during both experimental seasons (Tables S1 and S2). The magnitude of these reductions varied considerably among genotypes, reflecting strong genotype-dependent sensitivity. Heat stress represents a major constraint to global wheat production, particularly affecting key agronomic traits during reproductive and grain-filling stages (Ortiz-Bobea et al., 2019; Gautam et al., 2023; Pant et al., 2025).

Elevated temperatures, especially above 22 °C between anthesis and maturity, can decrease yield by 3–17% per 1 °C increase (Asseng et al., 2015; Pokhrel et al., 2019; Poudel et al., 2020). Wheat proves most sensitive during anthesis, where heat impairs pollen development, nutrient translocation, and anther dehiscence, leading to reduced pollen viability and seed set (Prasad Djanaguiraman, 2014; Djanaguiraman et al., 2020; Browne et al., 2021; Ullah et al., 2022). Temperatures exceeding 30 °C induce pollen sterility primarily through tapetum degeneration during microspore meiosis (Saini, Sedgley & Aspinall, 1984). Under heat stress in the 2020–2021 season, Meraj-08 and Gulzar-19 recorded PV values of 66%, while Ujala-15 declined to 62%; reductions intensified in 2021–2022, with Janbaz-09 and Ghazi-2019 at 57%, Sindhu-16 at 43%, and Manthar-2003 showing complete sterility (0%) (Saini, Sedgley & Aspinall, 1984; Prasad Djanaguiraman, 2014; Djanaguiraman et al., 2020).

A parallel decline occurred in grains per spike: Bahawalpur-97 recorded the lowest GpS (15) in 2020–2021, while Punjab-2011 (8) and Manthar-2003 (0) showed severe failure in 2021–2022 (Ferris et al., 1998; Farooq et al., 2011a; Farooq et al., 2011b; Farooq et al., 2011c; Mahboob et al., 2018; Hamam, Khaled & Zakaria, 2015; Shewry, 2009; Semenov, 2009; Modarresi et al., 2010; Gandahi et al., 2020; Alghabari et al., 2021). Spikelet fertility also dropped substantially, with Gold-16 at 55% in 2020–2021 and Pak-81 at 49.6% in 2021–2022; Manthar-2003 exhibited complete sterility (Kaur & Behl, 2010; Modarresi et al., 2010; Gandahi et al., 2020).

### Effects on grain filling and floret development

Grain filling duration shortened significantly under heat stress, with C-250 reduced to 15.66 days in 2020–2021 and both C-250 and Morocco at 18 days in 2021–2022 (Streck, 2005; Yin, Guo & Spiertz, 2009; Anjum et al., 2011; Sade et al., 2018). Fertile florets per spikelet reached a minimum of one floret in Morocco and LU-26 during 2020–2021, while NIFA-Aman, Shalimar-88, and Manthar-2003 showed zero in the second season. These patterns reflect genotype-specific reproductive failure above 30  °C, driven by disrupted fertilization and limited nitrogen/carbon availability (Stratonovitch & Semenov, 2015; Aiqing et al., 2018; Dwivedi et al., 2017). Reported GFD reductions include approximately 2.8 days per 1 °C increase and 12 days per 5 ° C increase, due to decreased assimilate supply and impaired enzymatic activity (Anjum et al., 2011; Sade et al., 2018; Liu et al., 2020; Arjona et al., 2020).

Anther length, a key determinant of male fertility, decreased to a minimum of 2.70 mm across 14 genotypes in 2020–2021 and 2.91 mm in Khushal-69 and Sutluj-86 in 2021–2022, impairing pollen production and grain set (Saini & Aspinall, 1982). Collectively, these results confirm that terminal heat stress severely disrupts wheat reproductive development, with pollen viability and associated traits as primary yield stability determinants under stress. Significant genetic variation for these traits under various conditions corroborates prior findings (Khan et al., 2021a; Khan et al., 2021b; Shenoda et al., 2021; Kumar et al., 2023; Ferris et al., 1998; Farooq et al., 2011a; Farooq et al., 2011b; Farooq et al., 2011c; Mahboob et al., 2018; Wahid et al., 2007).

### Trait associations under normal and heat stress conditions

Pearson’s correlation analysis revealed consistent and biologically meaningful associations among reproductive and yield-related traits of wheat under both normal and heat stress conditions across the two growing seasons (Table 3; Figs. S1 & S2). Under normal conditions in 2020–2021 (N1), pollen viability (PV) was positively correlated with grain number per spike (GpS; *r* = 0.21, *p* = 0.0028, **), spikelet fertility (SpF; *r* = 0.22, *p* = 0.0017, **), fertile florets per spikelet (FFpSp; *r* = 0.23, *p* = 0.0011, **) and anther length (AL; *r* = 0.16, *p* = 0.0236, *), with a weaker correlation observed for grain filling duration (GFD; *r* = 0.14, *p* = 0.0480, *). Grain number also showed strong positive associations with SpF (*r* = 0.41, *p* < 0.001, ***) and FFpSp (*r* = 0.14, *p* = 0.0480, *), indicating coordinated reproductive performance under optimal environments consistent with previous work showing linked reproductive trait responses in wheat (Saini & westgate, 2000; Impe et al., 2020; Masthigowda et al., 2022). Under normal conditions in 2021–2022 (N2), similar but weaker associations persisted, with significant PV–SpF (*r* = 0.15, *p* = 0.0340, *) and PV–AL (*r* = 0.21, *p* = 0.0028, **), and sustained GpS–SpF (*r* = 0.33, *p* < 0.0001, ***) and GpS–FFpSp (*r* = 0.17, *p* = 0.0161, *), reflecting stable trait interrelationships across seasons (Tshikunde et al., 2019; Bishnoi, Rana & Chaurasia, 2023).

**Table 3:** Pearson’s correlation coefficients among reproductive and yield-related traits in wheat under normal and heat stress conditions across two growing seasons (2020–2021 and 2021–2022). The table presents pairwise Pearson’s correlation coefficients among pollen viability (PV), grain number per spike (GpS), spikelet fertility (SpF), fertile florets per spikelet (FFpSp), grain filling duration (GFD), and anther length (AL). Correlations were calculated separately for each environment and season: N1 and N2 represent normal growing conditions during 2020–2021 and 2021–2022, respectively, while S1 and S2 represent heat stress conditions during the same seasons. Positive and negative values reflect the direction and strength of the associations among traits under different environmental conditions.

**Trait pair**	**N1 r (*p*-value)**	**N2 r (*p*-value)**	**S1 r (*p*-value)**	**S2 r (*p*-value)**
**PV–GpS**	0.21 ^**^ (0.0028)	0.06 ns (0.3987)	0.38 ^***^ (<0.0001)	0.43 ^***^ (<0.0001)
**PV–SpF**	0.22 ^**^ (0.0017)	0.15 ^*^ (0.0340)	0.30 ^***^ (<0.0001)	0.39 ^***^ (<0.0001)
**PV–AL**	0.16 ^*^ (0.0236)	0.21 ^**^ (0.0028)	0.13 ns (0.0665)	−0.14 ^*^ (0.0480)
**PV–GFD**	0.14 ^*^ (0.0480)	−0.06 ns (0.3987)	−0.08 ns (0.2601)	0.17 ^*^ (0.0161)
**PV–FFpSp**	0.23 ^**^ (0.0011)	−0.09 ns (0.2050)	0.10 ns (0.1589)	0.19 ^**^ (0.0070)
**GpS–GFD**	0.06 ns (0.3987)	0.02 ns (0.7786)	0.05 ns (0.4820)	0.01 ns (0.8882)
**GpS–SpF**	0.41 ^***^ (<0.0001)	0.33 ^***^ (<0.0001)	0.32 ^***^ (<0.0001)	0.28 ^***^ (0.0001)
**GpS–FFpSp**	0.14 ^*^ (0.0480)	0.17^*^ (0.0161)	0.12 ns (0.0905)	0.16 ^*^ (0.0236)
**GpS–AL**	0.00 ns (1.0000)	0.06 ns (0.3987)	–	–
**SpF–GFD**	−0.07 ns (0.3246)	0.06 ns (0.3987)	0.12 ns (0.0905)	0.31 ^***^ (<0.0001)
**SpF–FFpSp**	−0.02 ns (0.7786)	0.08 ns (0.2601)	0.04 ns (0.5739)	0.25 ^***^ (0.0004)
**SpF–AL**	0.08 ns (0.2601)	0.00 ns (1.0000)	0.06 ns (0.3987)	−0.05 ns (0.4820)
**FFpSp–GFD**	−0.02 ns (0.7786)	0.08 ns (0.2601)	0.04 ns (0.5739)	0.25 ^***^ (0.0004)
**FFpSp–AL**	0.01 ns (0.8882)	0.02 ns (0.7786)	−0.07 ns (0.3246)	0.02 ns (0.7786)
**SpF–FFpSp**	0.26 ^***^ (0.00018)	0.22^**^(0.0017)	0.17 ^*^(0.016)	0.23 ^**^ (0.0010)
**GFD–AL**	0.01 ns (0.8882)	−0.07 ns (0.3246)	–	–

**Notes.**

Statistical significance is indicated as ***, **, and * for *p* < 0.001, *p* < 0.01, and *p* < 0.05, respectively, and ns denotes non-significant correlations.

Under heat stress in 2020–2021 (S1), correlations among key reproductive traits were generally strengthened; PV was more strongly associated with GpS (*r* = 0.38, *p* < 0.0001, ***) and SpF (*r* = 0.30, *p* < 0.0001, ***), although its association with AL was not significant (*r* = 0.13, *p* = 0.0665, ns). However, GpS–SpF remained significant (*r* = 0.32, *p* < 0.0001, ***). In 2021–2022 (S2), PV–GpS (*r* = 0.43, *p* < 0.0001, ***) and PV–SpF (*r* = 0.39, *p* < 0.0001, ***) were again prominent, and moderate associations were observed for PV–FFpSp (*r* = 0.19, *p* = 0.0070, **), GpS–SpF (*r* = 0.28, *p* = 0.0001, ***) and GpS–FFpSp (*r* = 0.16, *p* = 0.0236, *). The persistence of positive correlations among PV, GpS, SpF, and FFpSp under heat aligns with reports that pollen viability closely relates to seed set and yield components under thermal stress in wheat and other cereals. (Cossani & Reynolds, 2012; Liu et al., 2019; Khan, Wu & Sajjad, 2022). In contrast, correlations involving GFD and AL tended to be weaker or non-significant across environments (Khan et al., 2024; Sakata & Higashitani, 2008; Riaz et al., 2021).

Overall, these results indicate that heat stress strengthens positive associations among reproductive traits, emphasizing the critical role of pollen viability and spike fertility in maintaining grain number under elevated temperatures—traits widely recognized as sensitive indicators of heat tolerance in wheat (Shenoda et al., 2021; Xu et al., 2022). This integrated trait network suggests that selection for improved PV and associated reproductive traits may enhance breeding for heat tolerance.

### Identification of heat-tolerant genotypes using heat susceptibility index

The heat susceptibility index (HSI) enabled classification of genotypes into tolerant, moderately tolerant, and susceptible groups across traits and seasons (Tables S3 & S4; Table 4). Across the two-year study, Chenab-70, Pak-81, and Frontana consistently exhibited low HSI values for pollen viability, grains per spike, spikelet fertility, and fertile florets per spikelet, indicating stable heat tolerance. In contrast, heat tolerance for grain filling duration was less consistent, with nine tolerant genotypes identified in the first year and only five in the second, highlighting the greater environmental sensitivity of GFD. This trait-specific variability underscores the importance of multi-trait selection strategies for breeding heat-resilient wheat cultivars (Devi et al., 2021; Lamba et al., 2023).

**Table 4:** The number of heat-tolerant genotypes selected on HSI indexes of the studied traits. Classification of wheat genotypes based on heat tolerance using the heat susceptibility index (HSI) for reproductive and yield-related traits. The table summarizes the number of genotypes identified as heat-tolerant for each trait, including pollen viability (PV), grain number per spike (GpS), spikelet fertility (SpF), fertile florets per spikelet (FFpSp), grain filling duration (GFD), and anther length (AL). Heat susceptibility indices (HSI) were calculated for each genotype based on performance under normal and heat stress conditions across two growing seasons (2020–2021 and 2021–2022). Lower HSI values indicate greater tolerance to heat stress. This classification provides a clear comparison of genotypic responses and highlights traits contributing most strongly to heat tolerance.

**Traits**	**Genotypes**	
	**2020–21 season (**HSI < 0.5**)**	**2021–22 season (**HSI < 0.5**)**
Pollen viability (PV)	68	84
Grains per spike (GpS)	12	17
Grain filling duration (GFD)	9	5
Spikelet fertility (SpF)	67	52
Fertile florets per spikelet (FFpSp)	14	25
Anther Length (AL)	0	31

### Genetic variability, heritability, and selection potential

Substantial genetic variability was observed for all traits under both normal and heat stress conditions across the two years (Tables 5 and 6). Grain number per spike (GpS) and anther length (AL) consistently showed high genotypic (GCV) and phenotypic coefficients of variation (PCV), with AL exhibiting identical GCV and PCV values in several environments, which indicates strong genetic control (Kumar et al., 2020a; Kumar et al., 2020b). Broad-sense heritability was high for pollen viability (PV), GpS, spikelet fertility (SpF), and AL under both conditions, coupled with high to moderate genetic advance, suggesting predominance of additive gene action, limited environmental influence, and high selection efficiency (Hossain et al., 2021; Almutairi, 2022; Krishna et al., 2023).

**Table 5:** Genetic parameters of the studied traits under normal and heat stress conditions during 2020–2021. Estimates of key genetic parameters for each trait, including genotypic variance (*σ*^2^g), phenotypic variance (*σ*^2^p), genotypic coefficient of variation (GCV), phenotypic coefficient of variation (PCV), broad-sense heritability (*H*^2^), and genetic advance as a percentage of the mean (GA%). Traits evaluated were pollen viability (PV), grain number per spike (GpS), spikelet fertility (SpF), fertile florets per spikelet (FFpSp), grain filling duration (GFD), and anther length (AL). Estimates were calculated separately for normal and heat stress environments during the 2020–2021 growing season. This information highlights the extent of genetic variability, heritability, and potential for selection of traits contributing to heat tolerance in wheat.

Traits	GCV (%) N1	GCV (%) S1	PCV (%) N1	PCV (%) S1	GA % mean (N1)	GA % mean (S1)	*h*^2^ (Broad sense)% (N1)	*h*^2^ (Broad sense)% (S1)
PV	3.6	7.3	3.7	7.4	7.0	14.9	91	98
GpS	20	23.2	21.03	24.3	39.5	45.5	91	91
GFD	8.2	10.8	11.8	15.5	11.7	15.6	48	49
SpF	4.6	8.6	4.9	10.4	8.9	14.8	87	69
FFpSp	15.4	14.2	34.5	43.4	14.2	9.6	19	11
AL	14.6	14.6	14.6	14.6	30.0	30.0	100	100

**Table 6:** Genetic parameters of the studied traits under normal and heat stress conditions during 2021–2022. The table presents estimates of key genetic parameters for each trait, including genotypic variance (*σ*^2^g), phenotypic variance (*σ*^2^p), genotypic coefficient of variation (GCV), phenotypic coefficient of variation (PCV), broad-sense heritability (H^2^), and genetic advance as a percentage of the mean (GA%). Evaluated traits include pollen viability (PV), grain number per spike (GpS), spikelet fertility (SpF), fertile florets per spikelet (FFpSp), grain filling duration (GFD), and anther length (AL). Estimates were calculated separately for normal and heat stress environments during the 2021–2022 growing season. These parameters indicate the extent of genetic variability, heritability, and potential response to selection, which are critical for improving heat tolerance in wheat.

Traits	GCV (%) N2	GCV (%) S2	PCV (%) N2	PCV (%) S2	GA % mean (N2)	GA % mean (S2)	h2 (Broad sense)% (N2)	h2 (Broad sense)% (S2)
PV	4.6	13.5	4.8	13.6	9.0	27.7	91	99
GpS	20.9	26.8	21.5	27.8	42.0	53.4	94	93
GFD	8.6	13.9	11.4	17	13.5	23.3	57	66
SpF	6.5	15.1	6.6	15.3	13.2	30.9	97	99
FFpSp	11.5	18.3	21.8	44.5	12.4	15.5	28	17
AL	7.3	10.2	7.3	10.2	15.0	20.9	100	100

In contrast, fertile florets per spikelet (FFpSp) displayed low heritability and low genetic advance, particularly under heat stress, indicating strong environmental modulation and reduced effectiveness of direct selection (Guo et al., 2020); Rehman et al., 2021). Grain filling duration (GFD) showed moderate broad-sense heritability (30–60%) with moderate genetic advance (10–20%) in the first year under both conditions, reflecting the involvement of both additive and non-additive gene effects (Plavšin et al., 2021). Although PV under normal conditions exhibited high heritability (>60%) across both years, it was accompanied by low genetic advance (<10%), signifying non-additive gene action and limited suitability for direct selection (Wan et al., 2022).

According to Johnson, Robinson & Comstock (1955), heritability is classified as low (<30%), medium (30–60%), or high (>60%), while genetic advance (as a percentage of the mean) is low (<10%), moderate (10–20%), or high (>20%). High heritability with high to moderate genetic advance for PV, GpS, and SpF under heat stress denotes that these traits are primarily governed by additive gene actions, making them promising targets for selection in breeding heat-tolerant wheat genotypes (Hossain et al., 2021; Almutairi, 2022; Krishna et al., 2023). The thermal stability and resilience of GpS and AL under heat stress further underscore their importance as key traits for genetic improvement (Reynolds, Braun & Cavalieri, 2010).

These patterns align with prior reports of low PCV and GCV for PV, GpS, and SpF across conditions, indicating minimal genetic variation and environmental influence (Lipi et al., 2020; Songsri et al., 2008). Heritability, as a measure of phenotypic variance attributable to genetic factors, serves a predictive role in crop improvement (Plavšin et al., 2021; Wan et al., 2022). The GpS and AL offer high potential for effective selection.

### SEM path analysis and principal component analysis of wheat yield traits

**Figure 1:**
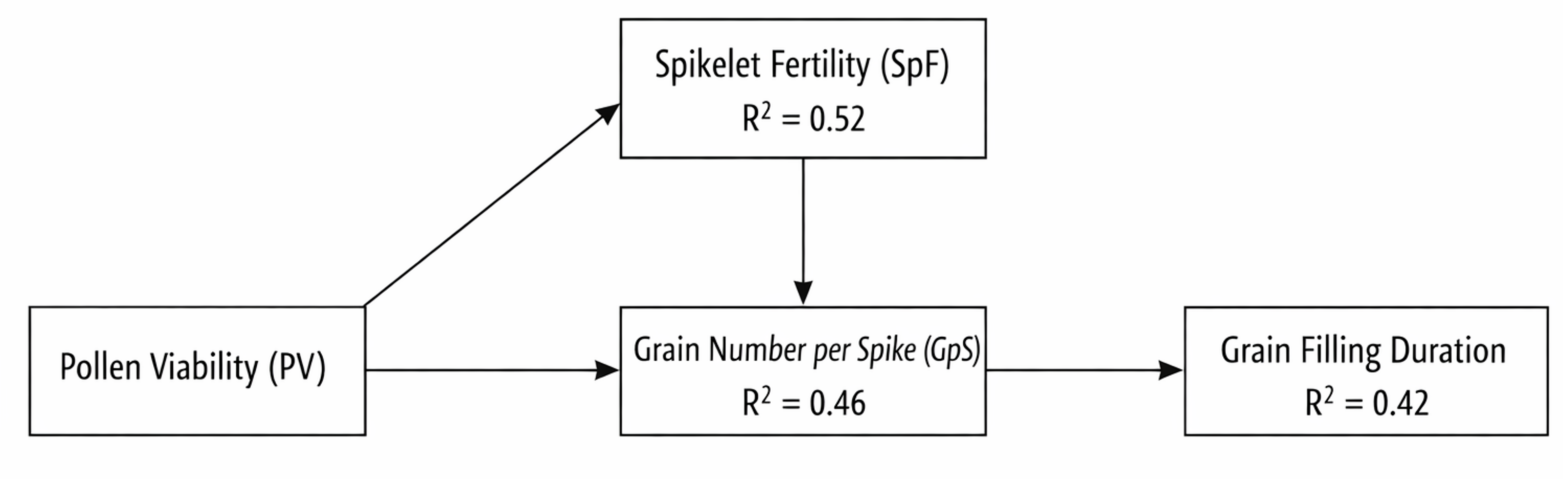
Structural equation model depicting the relationships among pollen viability, spikelet fertility, grain number per spike, and grain filling duration based on pooled data. Hypothesized causal relationships among traits based on pooled data, where pollen viability (PV) directly influences spikelet fertility (SpF) and grain number per spike (GpS). Spikelet fertility further contributes to variation in grain number per spike, which subsequently affects grain filling duration. Values inside boxes represent the coefficient of determination (*R*^2^) for each endogenous variable, indicating the proportion of variance explained by the model. Path coefficients were estimated using structural equation modeling (SEM), and only significant paths are presented (*P* < 0.05).

The structural equation model of the pooled dataset showed an acceptable fit to the data (*χ*^2^/*df* = 2.11, CFI = 0.95, TLI = 0.93, RMSEA = 0.048, SRMR = 0.041), indicating that the model reliably captured relationships among reproductive and yield traits. The SEM explained a moderate proportion of variance in key agronomic traits: grain number per spike (*R*^2^ = 0.46), spikelet fertility (*R*^2^ = 0.52), and grain filling duration (*R*^2^ = 0.42) (Fig. 1). These results indicate that the modeled associations account for a substantial portion of phenotypic variability (Table 7). Incremental fit indices confirmed good model performance, with CFI and TLI exceeding recommended thresholds, while RMSEA and SRMR values were within acceptable limits, consistent with established SEM fit criteria (Hu & Bentler, 1999). Collectively, these indices suggest that the SEM adequately represents the modeled associations among pollen viability, spikelet fertility, grain number per spike, and grain filling duration.

**Table 7:** Structural equation model (SEM) fit indices and proportion of variance explained (*R*^2^) for endogenous wheat traits based on pooled data across the 2020–2021 and 2021–2022 growing seasons. Fit indices reported include chi-square (*χ*^2^), degrees of freedom (df), chi-square to degrees of freedom ratio (*χ*^2^/df), comparative fit index (CFI), goodness-of- fit index (GFI), Tucker–Lewis index (TLI), and root mean square error of approximation (RMSEA). Criteria for acceptable model fit are *χ*^2^/df < 3, CFI, GFI, and TLI > 0.90, and RMSEA < 0.08. *R*^2^ values indicate the proportion of variance in each endogenous trait accounted for by the model, providing insight into the relative contribution of predictor variables in explaining variation under the experimental conditions.

**Measure**	**Value**	**Recommended threshold**	**Interpretation/variance explained**
**Fit indices**			
*χ*^2^/df	2.11	<3.0	Acceptable fit
CFI	0.95	>0.90	Good fit
TLI	0.93	>0.90	Good fit
RMSEA	0.048	<0.06	Good fit
SRMR	0.041	<0.08	Good fit
**Endogenous variables (R^2^)**			
Grain number per spike (GpS)	0.46	–	46% variance explained
Spikelet fertility (SpF)	0.52	–	52% variance explained
Grain filling duration (GFD)	0.42	–	42% variance explained

**Figure 2:**
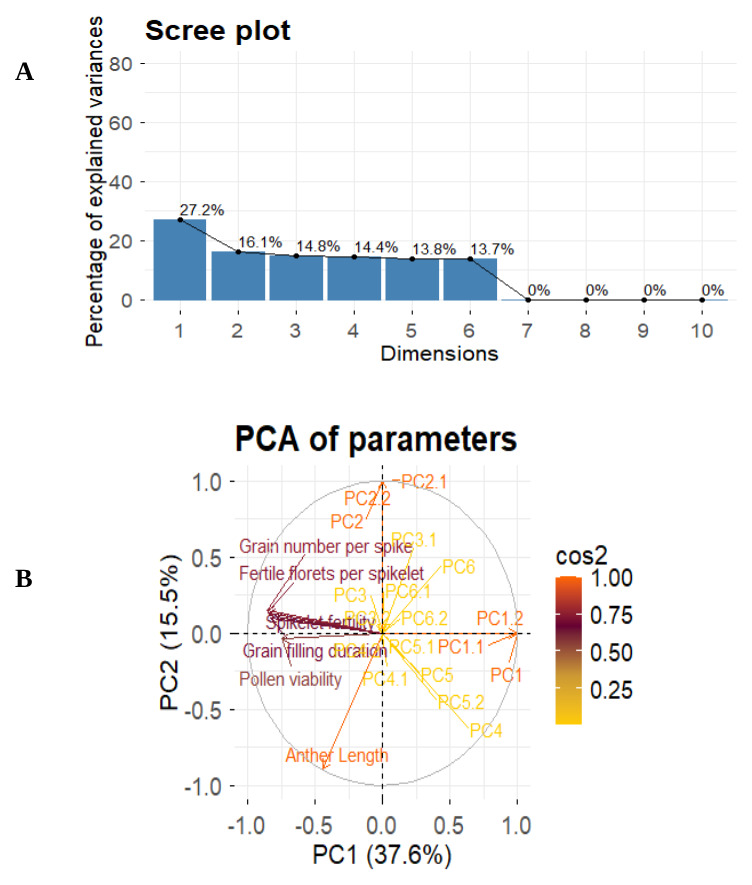
Principal component analysis (PCA) of 200 wheat genotypes under normal and heat stress conditions during 2020–2021. Principal component analysis (PCA) of reproductive and yield-related traits in wheat under normal and heat stress conditions (2020–2021). (A) Scree plot showing the proportion of total phenotypic variance explained by each principal component (PC), with emphasis on the leading PCs contributing most to overall variability. Trait loadings for pollen viability (PV), grain number per spike (GpS), spikelet fertility (SpF), fertile florets per spikelet (FFpSp), grain filling duration (GFD), and anther length (AL) are summarized to illustrate their relative contributions to multivariate variation under normal and heat stress environments. (B) PCA biplot depicting the relationships among the six traits and the multivariate distribution of wheat genotypes evaluated under normal and heat stress conditions. Vectors represent trait loadings, where vector length indicates the magnitude of contribution and the angle between vectors reflects the direction and strength of correlations among traits. Points represent individual wheat genotypes, with their spatial separation indicating differences in overall phenotypic performance across environments (2020–2021).

**Figure 3:**
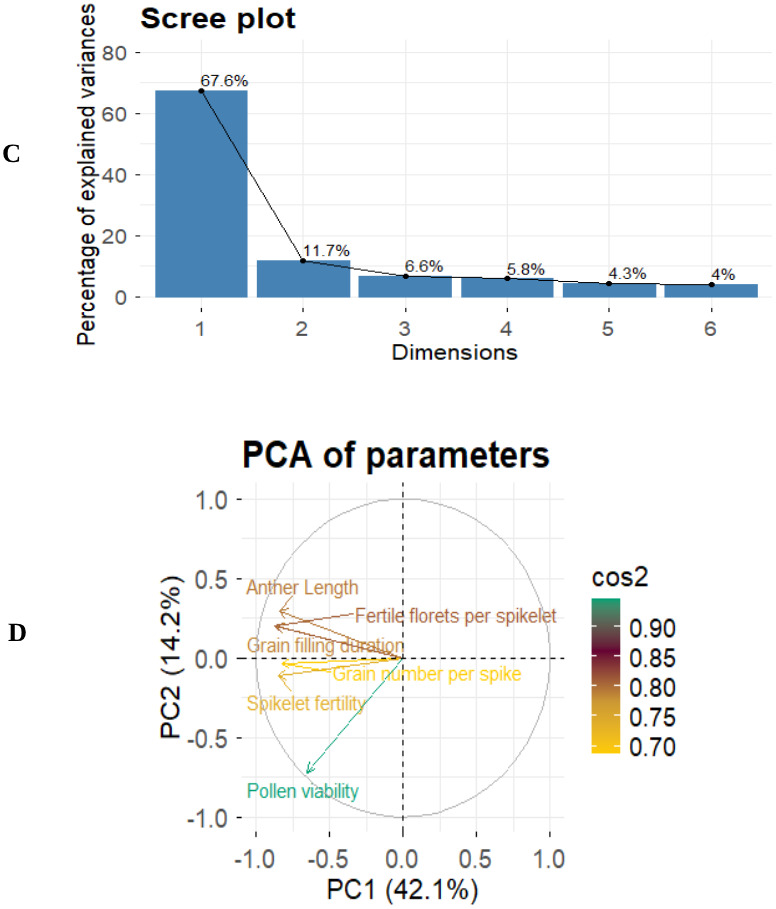
Principal component analysis (PCA) of 200 wheat genotypes under normal and heat stress conditions during 2021–2022. Principal component analysis (PCA) of reproductive and yield-related traits in wheat under normal and heat stress conditions during 2021–2022. (C) Scree plot showing the percentage of total phenotypic variance explained by each principal component (PC), highlighting the major PCs contributing to overall variability. Loadings of pollen viability (PV), grain number per spike (GpS), spikelet fertility (SpF), fertile florets per spikelet (FFpSp), grain filling duration (GFD), and anther length (AL) illustrate the relative contribution of each trait to multivariate variation under normal and heat stress environments. (D) PCA biplot illustrating trait associations and the multivariate distribution of wheat genotypes evaluated under normal and heat stress conditions. Trait vectors represent loadings on the principal components, where vector length indicates the magnitude of contribution and the angle between vectors reflects the direction and strength of correlations among traits. Individual points correspond to wheat genotypes, and their spatial separation reflects differences in overall phenotypic performance and response to heat stress during the 2021–2022 growing season.

These findings highlight the importance of male reproductive traits in relation to wheat yield under heat stress, consistent with earlier reports emphasizing pollen viability and anther development as critical upstream factors associated with spike fertility and grain filling (Farooq et al., 2011a; Farooq et al., 2011b; Farooq et al., 2011c; Talukder, McDonald & Gill, 2014; Zhao et al., 2022; Zahir et al., 2024). Maintaining high pollen viability and anther performance, therefore, appears to be associated with improved yield stability under elevated temperatures. Principal component analysis further supported these relationships by revealing distinct trait groupings across environments (Figs. 2 & 3). Under normal conditions, grain filling duration and grain number per spike clustered closely, reflecting coordinated developmental regulation (Zafar et al., 2020; Zhen et al., 2021), whereas under heat stress, pollen viability, spikelet fertility, and fertile florets per spikelet formed a tight cluster, suggesting a shared physiological response to thermal stress (Ahmad et al., 2025). These shifts emphasize the importance of reproductive traits in relation to heat tolerance, in agreement with previous studies (Talukder, McDonald & Gill, 2014; Zafar et al., 2020; Shokat, Sehgal & Vikram, 2021; Das et al., 2024; Kumar et al., 2025).

The SEM explained a moderate proportion of variance in key agronomic traits, indicating that the modeled associations account for substantial phenotypic variability, although additional genetic and environmental factors not included in the model may also contribute. Importantly, these pathways should be interpreted as statistical associations rather than definitive causal relationships because SEM was applied to observational field data rather than controlled experiments (Grace, 2006; Kline, 2016). Nevertheless, the consistency of SEM and PCA results supports the importance of reproductive traits in grain formation and yield stability under stress conditions. The final SEM diagram (Fig. 1) illustrates hypothesized direct and indirect pathways among traits with R^2^ values for variables, providing a visual framework for integrating agronomic traits and guiding future breeding strategies aimed at improving yield stability under variable environmental conditions.

### Implications for wheat breeding under heat stress

Overall, the integrated analyses demonstrate that pollen viability, grains per spike, spikelet fertility, and anther length are key determinants of heat tolerance in wheat. Their strong genetic control, stable correlations, and significant contributions to yield-related traits make them valuable selection criteria for breeding programs targeting terminal heat stress. The identified heat-tolerant genotypes provide promising genetic resources for developing resilient wheat cultivars adapted to rising temperatures.

## Conclusion

This study revealed substantial genetic variability among wheat genotypes for reproductive and yield-related traits under normal and heat stress conditions, with strong genotype × environment interactions. Heat stress significantly reduced pollen viability, grains per spike, spikelet fertility, grain filling duration, fertile florets per spikelet, and anther length, highlighting the sensitivity of reproductive processes during anthesis. Integrated analyses indicated that pollen viability, anther length, and fertile florets per spikelet are key associated determinants of grain number and spikelet fertility, emphasizing the role of male reproductive resilience in maintaining yield under high temperatures. High heritability and genetic advance for pollen viability, grains per spike, spikelet fertility, and anther length suggest effective selection potential, while traits such as fertile florets per spikelet and grain filling duration were environmentally sensitive.

The heat susceptibility index identified Chenab-70, Pak-81, and Frontana as consistently heat-tolerant across seasons, providing valuable germplasm for breeding. However, as this study was conducted at a single location (NARC, Islamabad), multi-location validation will be essential for broader generalization.

## Supplemental Information

10.7717/peerj.21335/supp-1 Supplemental Information 1 Supplemental Tables

10.7717/peerj.21335/supp-2 Supplemental Information 2 Correlation analysis between pollen viability and yield-related traits in 200 spring wheat genotypes under normal conditions during 2020–2021 and 2021–2022 Correlation plots showing pairwise relationships between pollen viability (PV) and yield-related traits, including grain number per spike (GpS), spikelet fertility (SpF), fertile florets per spikelet (FFpSp), anther length (AL), and grain filling duration (GFD) under normal growth conditions. (a) Correlation structure observed during the 2020–2021 growing season; (b) correlation structure observed during the 2021–2022 growing season. The color intensity and size of the circles indicate the magnitude and direction of Pearson’s correlation coefficients (r), where larger and darker circles represent stronger positive or negative correlations.

10.7717/peerj.21335/supp-3 Supplemental Information 3 Correlation analysis between pollen viability and yield-related traits in 200 spring wheat genotypes under heat stress conditions during 2020–2021 and 2021–2022 Correlation plots showing pairwise relationships between pollen viability (PV) and yield-related traits, including grain number per spike (GpS), spikelet fertility (SpF), fertile florets per spikelet (FFpSp), anther length (AL), and grain filling duration (GFD) under heat stress conditions. (c) Correlation structure observed during the 2020–2021 growing season; (d) correlation structure observed during the 2021–2022 growing season. The color intensity and size of the circles indicate the magnitude and direction of Pearson’s correlation coefficients (r), with larger and darker circles representing stronger positive or negative correlations.

## Additional Information and Declarations

### Data Availability

The following information was supplied regarding data availability: The raw data is available in the Supplemental Files.
