# Supplementary figures and images for: Trait prioritization and genotype selection for heat stress tolerance in wheat via structural equation modeling and principal component analysis (PCA)
Source: PeerJ. 2026 Jun 23;14:e21335. doi: 10.7717/peerj.21335 (PMC13308541; doi:10.7717/peerj.21335)

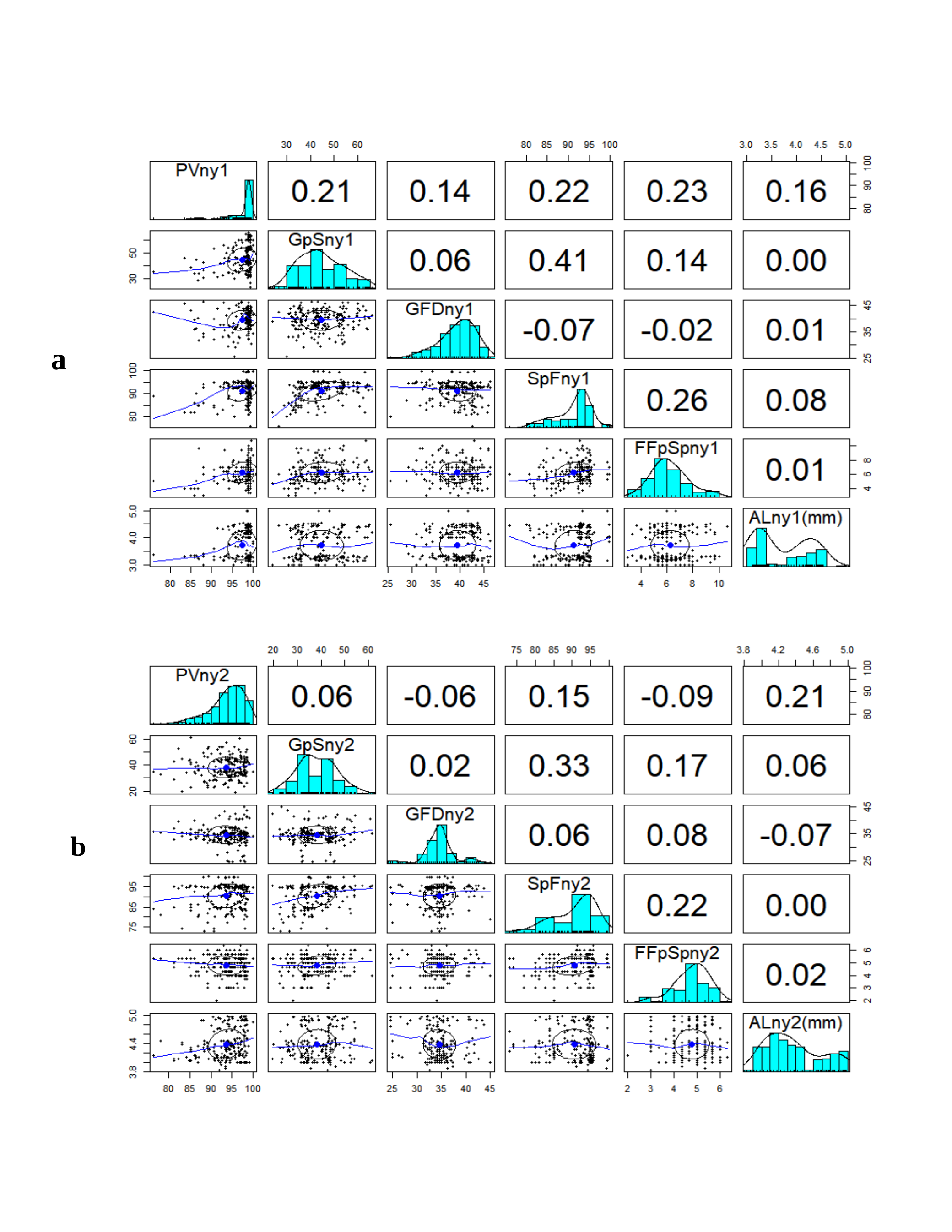

Supplement: Supplemental Information 2 — Correlation plots showing pairwise relationships between pollen viability (PV) and yield-related traits, including grain number per spike (GpS), spikelet fertility (SpF), fertile florets per spikelet (FFpSp), anther length (AL), and grain filling duration (GFD) under normal growth conditions. (a) Correlation structure observed during the 2020–2021 growing season; (b) correlation structure observed during the 2021–2022 growing season. The color intensity and size of the circles indicate the magnitude and direction of Pearson’s correlation coefficients (r), where larger and darker circles represent stronger positive or negative correlations. [file peerj-14-21335-s002.png]

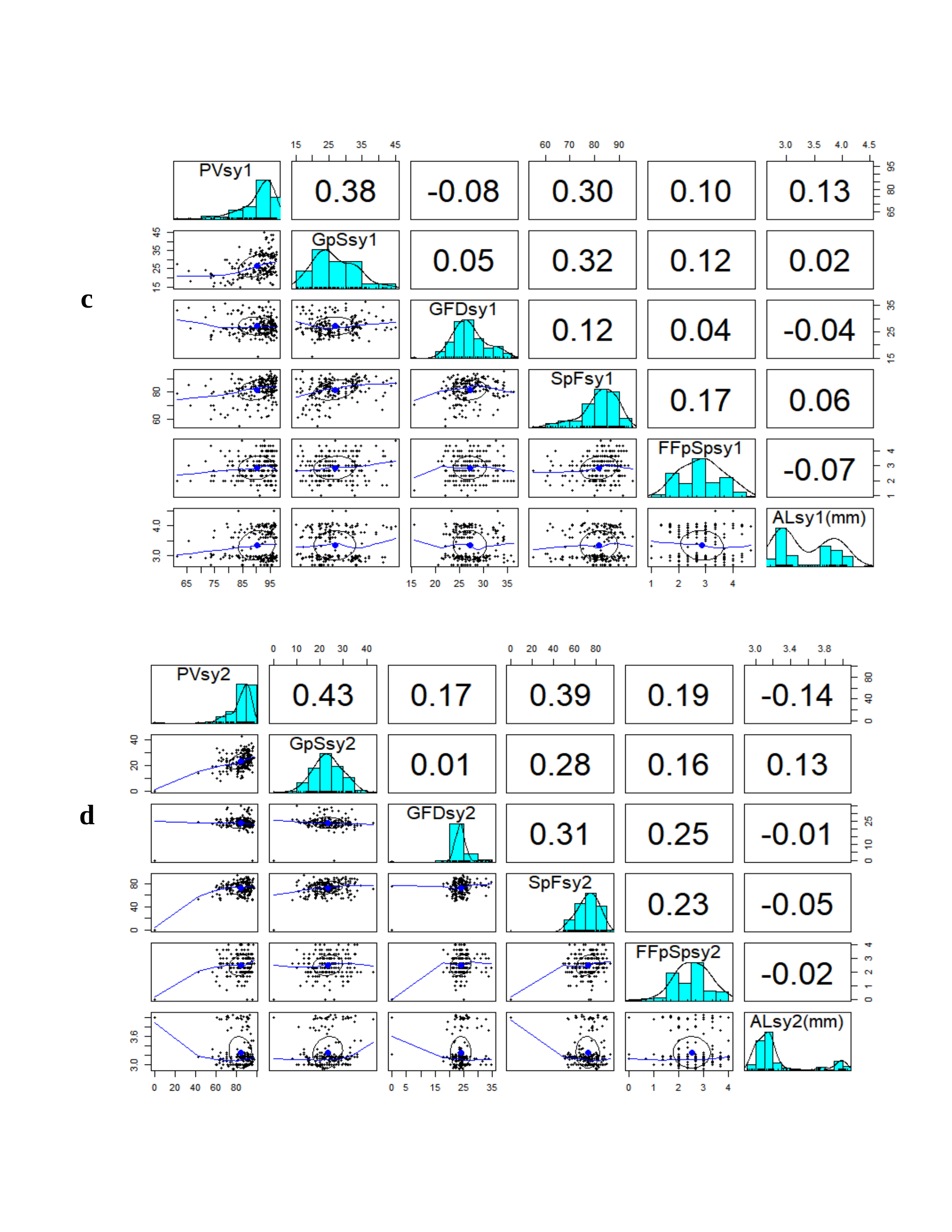

Supplement: Supplemental Information 3 — Correlation plots showing pairwise relationships between pollen viability (PV) and yield-related traits, including grain number per spike (GpS), spikelet fertility (SpF), fertile florets per spikelet (FFpSp), anther length (AL), and grain filling duration (GFD) under heat stress conditions. (c) Correlation structure observed during the 2020–2021 growing season; (d) correlation structure observed during the 2021–2022 growing season. The color intensity and size of the circles indicate the magnitude and direction of Pearson’s correlation coefficients (r), with larger and darker circles representing stronger positive or negative correlations. [file peerj-14-21335-s003.png]
